# Optical Fiber Methane Sensor Based on Mach–Zehnder Interferometer Induced by Multimode Interference

**DOI:** 10.3390/mi16040406

**Published:** 2025-03-29

**Authors:** Fuling Yang, Sicheng Zong, Xinghan Li, Yating Hu, Zelong Wang, Yuanyuan Qu, Jing Wang, Yan Li

**Affiliations:** School of Mechanical and Electrical Engineering, China University of Mining and Technology-Beijing, Beijing 100083, China; flyang@cumtb.edu.cn (F.Y.); sczong33@163.com (S.Z.); lixinghan1996@126.com (X.L.); 15160219690@163.com (Y.H.); 18744100883@163.com (Z.W.); 201419@cumtb.edu.cn (Y.Q.); 050441jj@163.com (J.W.)

**Keywords:** methane sensor, Mach–Zehnder interferometer, optical fiber, multimode interference

## Abstract

In this paper, based on the multimode interference structure fiber and the sensitive advantages of a zeolitic imidazolate framework-8/Polydimethylsiloxane (ZIF-8/PDMS)-sensitive film in methane detection, a methane sensor based on an interferometer induced by multimode interference is designed and built with the aid of modeling. The methane-sensitive single mode fiber (MS-SMF) is obtained by coating a ZIF-8/PDMS-sensitive film around the cladding of a thin-diameter SMF. The change in methane concentration leads to a change in the cladding mode of the MS-SMF, which causes a change in interference spectrum and realizes methane concentration sensing. The factors affecting the sensitivity of the methane sensor are analyzed. Methane sensors with various parameters are fabricated and tested on a methane sensor platform for performance estimation at methane concentrations of 0–4%. The experimental results show that the sensitivity of the sensor to methane reaches 2.364 nm/% when the length of the MS-SMF is 42 mm, the thickness of the sensitive film is 1.8 µm, and the diameter of the MS-SMF is 58 µm. The limit of detection is about 338 ppm. The average response time is 30 s and the recovery time is 45 s. The temperature sensitivity of the methane sensor is approximately 0.026 nm/°C. The experimental results verify the correctness of the methane sensor model. This study provides a new design idea for optical methane sensors, showing great application potential in the field of methane detection.

## 1. Introduction

Gas explosions are a major disaster in coal mining [1]. Methane, the primary component of coal mine gas, has an explosive concentration range of 5–15% in air [2]. In mining operations, the methane concentration must be strictly controlled within safe limits to prevent catastrophic accidents, making high-sensitivity and rapid methane detection critically important [3,4,5]. Traditional methane detection methods primarily include resistive [6,7,8] and electroacoustic sensors [9,10,11]; however, their electrical characteristics inherently pose potential explosion risks when detecting flammable and explosive gases. In recent decades, optical fiber methane sensors have gained significant attention in methane detection due to their intrinsic safety (eliminating electrical sparks in flammable environments), remote monitoring capability, strong resistance to electromagnetic interference, and non-invasive measurement, demonstrating promising application prospects [12,13,14,15].

Optical fiber methane sensors primarily operate on optical principles, detecting methane concentration through variations in light absorption and refractive index induced by methane [16,17,18]. These sensors have various structures, including surface plasmon resonance (SPR) optical fiber sensors [19,20], long-period grating (LPG) sensors [21,22], photonic crystal fiber (PCF) sensors [23,24], and multimode interference (MMI) sensors [25]. While these sensors have achieved methane detection in the range of 0–3.5%, the limit of detection (LOD) is not better than 500 ppm, and the sensitivity of most experimental tests do not reach 2 nm/%. Additionally, their response times (tens of seconds to minutes) hinder real-time monitoring and explosion warning. Among them, MMI-based optical fiber sensors utilize multimode transmission and interference effects to achieve sensitive responses to environmental variation. Their simple structure, high sensitivity, and fast response provide technical support for improving the performance of methane sensors [26,27,28].

The optical fiber device is not selective to methane, but the sensitive film coated on the optical fiber surface is selective to methane. Therefore, the design and fabrication of a suitable methane-sensitive film is the key to the production of high-performance methane sensors. Methane-sensing materials mainly include metal oxides [29], carbon materials [30], conductive polymers [31], supramolecules (Cryptophanes) [32], and metal-organic frameworks (MOFs) [33]. While some methane sensors based on metal oxides often show relatively good selectivity, they often require high temperature conditions, which are undesirable for detecting flammable and explosive gases that pose a risk of explosion [34,35]. Carbon materials sense methane based on changes in electrical or optical properties. However, theoretical and experimental studies have shown that sensors based on raw carbon materials have poor sensitivity and selectivity for methane [36,37]. Therefore, carbon materials are often modified using functional groups, dopants, metal nanoparticles, metal oxide nanoparticles, and polymers to overcome methane detecting limitations, which increases the cost and difficulty of material preparation [38,39]. Conductive polymers can sense methane based on changes in electrical, mass, or optical properties. The properties of conductive polymers are largely determined by their doping levels, which require chemical reactions with many analytes to change, increasing the complexity of material preparation and easily affecting the selectivity of methane [40,41]. Cryptophanes can sense methane based on changes in mass or optical properties. However, Cryptophanes usually need to be stored in dry conditions to maintain their properties [42,43], which is not conducive to their application in mine environments with a wide humidity range. MOF materials sense methane based on mass, optics, or electricity [44,45]. As an MOF material, zeolitic imidazolate framework-8 (ZIF-8) is an ideal methane-sensitive material with high porosity, excellent methane adsorption properties, and good chemical stability [46,47,48,49]. Polydimethylsiloxane (PDMS) has good methane permeability and stable mechanical properties [50,51,52], which can provide ideal support and protection for ZIF-8. Therefore, the composite film composed of ZIF-8 and PDMS can realize the reversible and rapid response to methane at room temperature based on the mechanism of physical adsorption.

In this paper, an optical fiber methane sensor based on a Mach–Zehnder interferometer (MZI) induced by MMI is designed. The methane-sensitive single mode fiber (MS-SMF) in the sensor is obtained by coating a ZIF-8/PDMS-sensitive film around the cladding of a fine-diameter SMF. By establishing the model of the relationship between methane concentration and MMI, the working principle and influencing factors of the sensor are revealed. The structural parameters of the sensor are designed by numerical analysis and optical simulation software, and the effects of the length of the MS-SMF, the diameter of the MS-SMF, and the thickness of the sensitive film on the sensitivity of the sensor are analyzed. In the aspect of sensor fabrication, the MMI structure is fabricated by fusion and splicing technology, and the ZIF-8/PDMS-sensitive film is coated on the fiber surface by the lift dip coating method. Finally, the methane-sensing experimental platform is established to conduct an experimental validation of the sensor’s performance. This study not only provides a new idea and method for the field of methane sensing, but also provides a useful reference for the design of other gas sensors.

## 2. Sensor Modeling and Design

### 2.1. Sensing Mechanism Modeling

**Figure 1:**
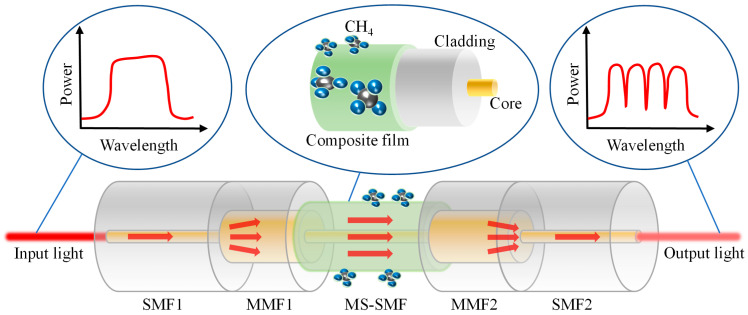
Schematic structure of multimode interference methane sensor based on ZIF-8/PDMS-sensitive film.

The structure diagram of the designed methane sensor based on MZI induced by multimode interference is shown in Figure 1. Its sensing structure is mainly combined with the multimode interference fiber sensor and ZIF-8/PDMS composite membrane. When light enters multimode fiber 1 (MMF_1_) from SMF_1_, multiple modes of MMF_1_ are excited due to a large core diameter mismatch. Each mode has a different propagation constant. In these modes, the ones with higher orders that are excited are dominant, and with a certain phase difference in between. When light enters the MS-SMF from MMF_1_, these modes and their respective phases are coupled back into the MS-SMF, resulting in a periodic interference spectrum, the period of which is determined by the cumulative phase difference between the MS-SMF modes. In this case, the higher-order modes that are excited mainly exist in the MS-SMF cladding, which is sensitive to external parameter changes. The ZIF-8/PDMS-sensitive film is coated around the MS-SMF cladding. The refractive index of the ZIF-8/PDMS-sensitive film will decrease after the adsorption of methane, and the new cladding composed of the ZIF-8/PDMS-sensitive film and MS-SMF cladding will be affected by the methane concentration in the environment, thus affecting the interference of excited high-order modes in the MS-SMF. When light enters MMF_2_ from the MS-SMF, the MMF_2_ acts as a beam synthesizer, coupling the higher-order modes that are excited and the fundamental mode to the SMF_2_. Ignoring the transmission loss of optical fiber, the intensity of the interference light can be expressed as follows:

(1) $$I=I_{core}+I_{cladding}+2\sqrt{I_{core}I_{cladding}}\cos\left(\Delta \varphi \right)$$

where *I_core_* and *I_cladding_* are light intensities in the MS-SMF core and cladding, respectively. Δ*φ* is the phase difference between the core mode light and the cladding mode light, which can be expressed as follows:

(2) $$\Delta \varphi =\frac{2\pi }{\lambda }\left(n_{co-eff}-n_{cl-eff}\right)L$$

where *λ* is the wavelength of the input light, and *n*_*co*−*eff*_ and *n*_*cl*−*eff*_ are the effective mode indices of the core mode and the cladding mode, respectively. *L* is the length of the MS-SMF. When the phase difference is an odd multiple of *π*, the light interference intensity in the core mode and the cladding mode is minimal, and the corresponding wavelength can be expressed as follows:

(3) $$\lambda_{m}=\frac{2L}{2m+1}\left(n_{co-eff}-n_{cl-eff}\right)$$

where *m* is the interference order.

The free spectral range (*FSR*) of interferometric spectra can be expressed as follows:

(4) $$FSR=\lambda_{m}-\lambda_{m+1}=\frac{\lambda_{m}\lambda_{m+1}}{\left(n_{co-eff}-n_{cl-eff}\right)L}$$

When the refractive index of the ZIF-8/PDMS-sensitive film changes due to the change in the methane concentration in the environment, the corresponding wavelength shift can be expressed as follows:

(5) $$\Delta \lambda_{m}=\frac{2L}{2m+1}\left(n_{co-eff}-\frac{𝜕n_{cl-eff}}{𝜕n_{cf-eff}}\frac{𝜕n_{cf-eff}}{𝜕C_{CH_{4}}}\Delta C_{CH_{4}}\right)$$

where 𝜕*n*_*cl*−*eff*_/𝜕*n*_*cf*−*eff*_ and 𝜕*n*_*cf*−*eff*_/𝜕*C_CH_*_4_ represent the influence coefficient of the ZIF-8/PDMS-sensitive film effective index (*n*_*cf*−*eff*_) on that of the cladding mode, and the influence coefficient of the methane concentration (*C_CH_*_4_) on the ZIF-8/PDMS-sensitive film effective index, respectively; Δ*C_CH_*_4_ represents the change in the methane concentration in the environment. When the concentration of methane in the environment increases, the effective index of the ZIF-8/PDMS-sensitive film will decrease; consequently, the effective index of the cladding mode will decrease, and the effective index difference between the core mode and the cladding mode will increase accordingly. As a result, the wavelength of the valley will be redshifted. Thus, the concentration of methane in the environment can be obtained by observing the wavelength shifts of the spectra valley. The sensitivity of the sensor can be expressed as follows:

(6) $$S=\frac{d\lambda_{m}}{dC_{CH_{4}}}=-\frac{2L}{2m+1}\frac{𝜕n_{cladding}}{𝜕n_{ZIF-8}}\frac{𝜕n_{ZIF-8}}{𝜕C_{CH_{4}}}$$

From Equation (6), it is obvious that there are three factors affect the methane sensitivity: the length of the MS-SMF, the thickness of ZIF-8/PDMS-sensitive film on the methane adsorption capacity, and the influence of the MS-SMF diameter on the cladding recombination mode.

### 2.2. Sensor Structure Design

According to the sensing model of methane sensor, the influence of various structural parameters on the sensitivity of the methane sensor is analyzed by simulation. When making the sensor, it can provide the basis for the design of structural parameters, and then obtain reliable sensing performance. The parameters of the simulation model are set as shown in Table 1. The size parameters of the interference structure will affect the FSR and contrast of the interference spectrum. The FSR and contrast of the interference spectrum will affect the demodulation accuracy of the interference trough and thus affect the performance of the sensor. Therefore, in the selection of structural parameters, we first set the sensor structure size according to the requirements of the project for the installation size of the sensor application. Then, we refer to the references of MMI fiber optic sensors and further limited the sensor structure size. Finally, the optical fiber sizes that can be manufactured are discussed with the optical fiber manufacturers, and the parameters of the simulation structure are selected on this basis. Through the FSR and contrast of the interference spectrum, suitable parameters are selected for simulation.

**Figure 2:**
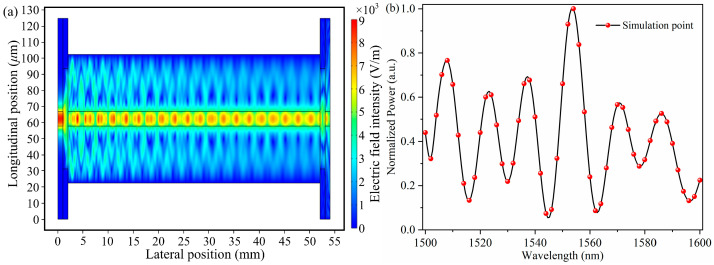
(**a**) Simulation of optical field distribution for cross-sectional optical path in MMI structure; (**b**) simulation of interference spectrum for MMI structure.

The simulation results of the optical field transmission and diffusion of this structure are shown in Figure 2a. It can be seen from the simulation results that when the light is coupled from the SMF_1_ to the MMF_1_, the light energy from the fiber core decreases rapidly, which means that multiple high-order modes are excited from the SMF_1_, and these modes exist in the MMF_1_ and interfere with each other, resulting in the redistribution of the optical field energy. The interference spectrum of the MMI structure is shown in Figure 2b. It can be seen that the core mode and cladding mode in the structure have interference, and the interference intensity corresponding to different incident wavelengths is different.

In order to obtain the response characteristics of the refractive index of the ZIF-8/PDMS-sensitive film to the concentration of methane, a refractometer is used to measure the refractive index of the sensitive film with the concentration of methane ranging from 0% to 4% firstly. The refractive index (*n*_film_) of the sensitive film decreases linearly with the increase in the methane concentration (*C*_CH4_), *n*_film_ = 1.4143–0.0031*C*_CH4_. Then, the ZIF-8/PDMS-sensitive film and the MS-SMF cladding are equivalent to the composite cladding. According to the size and refractive index parameters of the MS-SMF and ZIF-8/PDMS-sensitive films, the equivalent refractive index of the composite cladding can be obtained by finite element analysis. The effective index of the composite cladding is used as the effective index of the cladding mode (*n*_*cl*−*eff*_) in Equation (3), and the interference spectrum of the sensor is obtained by combining Equations (1) and (2). The wavelength shift of the interference valley when the per unit methane concentration changes represents the sensitivity of the multimode interference methane sensor.

**Figure 3:**
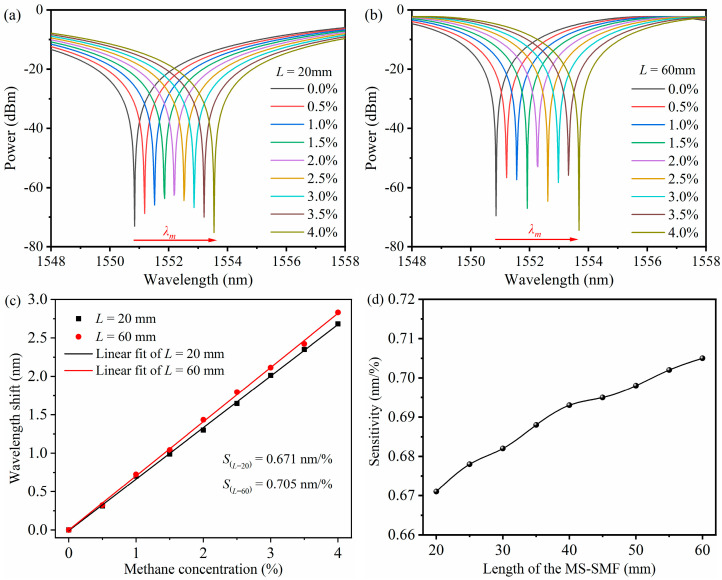
(**a**) Simulation results of interference spectrum of the sensor at 0–4% methane concentration when the length of MS-SMF is 20 mm. (**b**) Simulation results of interference spectrum of the sensor at 0–4% methane concentration when the length of MS-SMF is 60 mm. (**c**) Linear fitting results of interference valley shifts as methane concentration changes when the length of MS-SMF is 20 mm and 60 mm. respectively. (**d**) Relationship between sensitivity of methane sensor and MS-SMF length.

Based on the design requirements of sensor miniaturization, the size of the whole methane sensor should not be too large, and it is required to present several interference spectrum periods in the working band of a wide spectrum light source, so as to capture and measure the dynamic change in the wavelength of the interference valley within one order. The interference valley near 1550 nm is selected as the indicating wavelength. Through a large number of simulations, the length of the MS-SMF (*L*) is in the range of 20–60 mm, and the number and period of interference valleys are suitable. As shown in Figure 3a,b, when the thickness of the sensitive film is 0.5 μm, the diameter of the MS-SMF is 100 μm, and the length of the MS-SMF is 20 mm and 60 mm, respectively, the interference valley shifts with the methane concentration. When the length of the MS-SMF is 20 mm and 60 mm, respectively, and the methane concentration in the environment rises from 0 to 4%, the valley of the interference spectrum is redshifted. The slope obtained by fitting the data with the least square method indicates the sensitivity of the sensor, as shown in Figure 3c. Therefore, the sensitivity of the sensor is 0.671 nm/% and 0.705 nm/%, respectively. The influence of the MS-SMF length on methane sensor sensitivity is shown in Figure 3d. In the range of 1530–1600 nm, there are multiple valleys in the interference spectrum, and different valleys correspond to a different interference order *m*. When different interference valleys are selected as the indicator wavelength of the sensor, the sensitivity of the sensor will have little change. The sensitivity is slightly higher at long indicator wavelengths. Therefore, the sensitivity of the sensor is mainly affected by *L*/*m* and has little relationship with the length of the MS-SMF. However, the length of the MS-SMF will affect the period and mechanical stability of the interference spectrum. Considering the above factors, the length of the MS-SMF is set to 40 mm.

**Figure 4:**
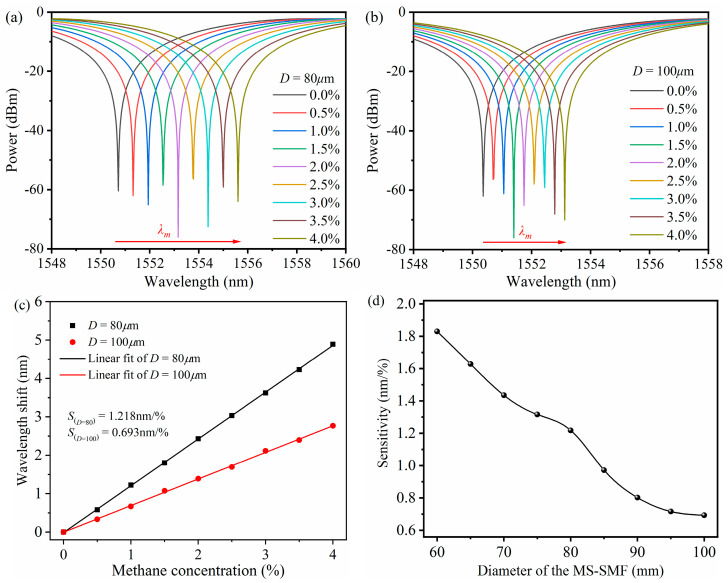
(**a**) Simulation results of interference spectrum of the sensor at 0–4% methane concentration when the diameter of the MS-SMF is 80 mm. (**b**) Simulation results of interference spectrum of the sensor at 0–4% methane concentration when the diameter of the MS-SMF is 100 mm. (**c**) Linear fitting results of interference valley shifts as methane concentration changes when the diameter of the MS-SMF is 80 mm and 100 mm, respectively. (**d**) Relationship between sensitivity of methane sensor and MS-SMF diameter.

Figure 4a,b shows the interference spectrum of the interference valley with the sensor at a 0–4% methane concentration when the thickness of the sensitive film is 0.5 μm, the length of the MS-SMF is 40 mm, and the diameter of the MS-SMF is 80 μm and 100 μm, respectively. Figure 4c shows the relationship between interference valley shifts and methane concentration, and the wavelength shift increases approximately linearly with the increase in the methane concentration. The sensitivity of the multimode interference methane sensor based on the ZIF-8/PDMS-sensitive film is 1.218 nm/% and 0.693 nm/%, respectively. The influence of the MS-SMF diameter on methane sensor sensitivity is shown in Figure 4d. The simulation results show that the sensitivity of the multimode interference methane sensor based on the ZIF-8/PDMS-sensitive film increases with the decrease in the MS-SMF diameter.

**Figure 5:**
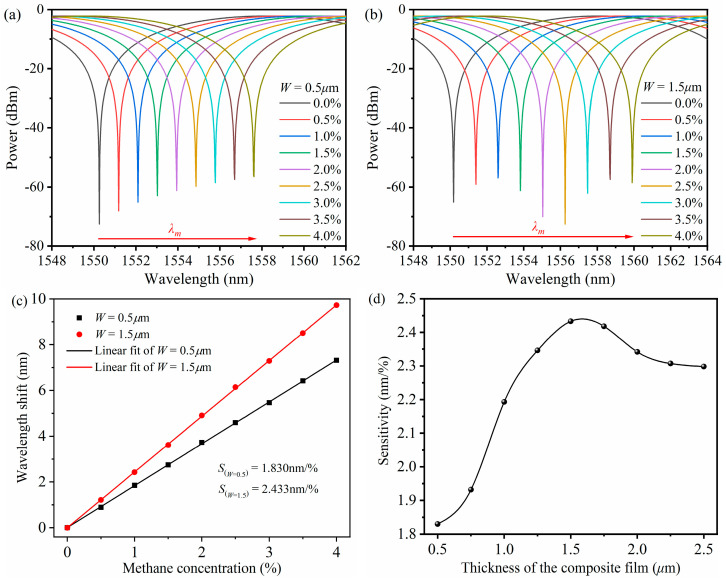
(**a**) Simulation results of interference spectrum of the sensor at 0–4% methane concentration when the thickness of the sensitive film is 0.5 μm. (**b**) Simulation results of interference spectrum of the sensor at 0–4% methane concentration when the thickness of the sensitive film is 1.5 μm. (**c**) Linear fitting results of interference valley shifts as methane concentration changes when the thickness of sensitive film is 0.5 μm and 1.5 μm, respectively. (**d**) Relationship between sensitivity of methane sensor and sensitive film thickness.

Considering the diffusion effect of methane on the surface of the ZIF-8/PDMS-sensitive film, the sensitive film thickness (*W*) is set to be 0.5 μm, 1.0 μm, and 1.5 μm, respectively. As can be seen from Equation (6), when the surface thickness of the composite layer increases, the methane molecules absorbed by ZIF-8 in the sensitive film will increase, the cladding mode excitation in the MS-SMF will intensify, the effective index difference between the cladding mode and the core mode will increase, and the wavelength shift will increase; thus, the sensitivity will be improved. Figure 5a,b show the interference spectrum of the multimode interference methane sensor based on the ZIF-8/PDMS-sensitive film with a methane concentration of 0–4% when the sensitive film thickness is 0.5 μm and 1.5 μm, respectively. Figure 5c shows the relationship between the interference valley shift and methane concentration, and the corresponding wavelength shift increases approximately linearly with the increase in the methane concentration. The sensitivity of the multimode interference methane sensor based on the ZIF-8/PDMS-sensitive film is 1.830 nm/% and 2.433 nm/%, respectively. The influence of the sensitive film thickness on methane sensor sensitivity is shown in Figure 5d. The simulation results show that when the thickness of the sensitive film is less than 1.6 μm, the sensitivity of the multimode interference methane sensor increases with the increase in the sensitive film thickness. When the thickness of the sensitive film is greater than 1.6 μm, an excessively thick sensitive film will weaken the influence of the methane adsorption sensitive film on the cladding mode, resulting in a decrease and stabilization of the multimode interference methane sensor sensitivity with an increasing sensitive film thickness.

## 3. Experimental Results and Discussion

### 3.1. Sensor Fabrication and Experimental System

**Figure 6:**
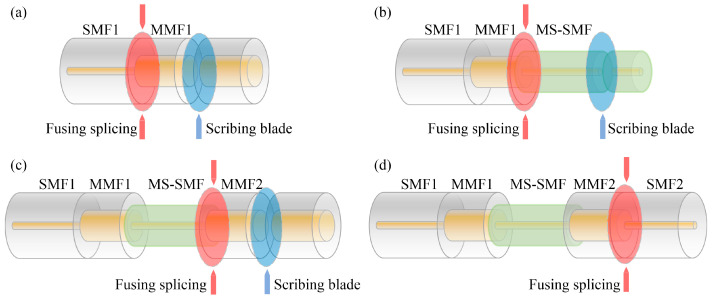
Fabrication process of multimode interference structure fiber. (**a**) Splice SMF1 with MMF1 and cut off MMF1 to a desired length. (**b**) Prepare a section of MS-SMF coated with ZIF-8/PDMS sensitive film and splice it with the MMF1. (**c**) Connect the cleaved MS-SMF with MMF2 of specified length. (**d**) Cascade SMF2 with MMF2.

**Figure 7:**
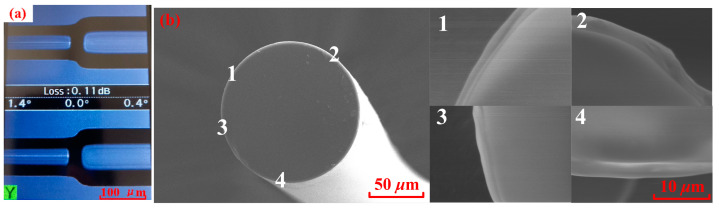
(**a**) Fusion of MS-SMF and MMF. (**b**) SEM image of cross-section of coated fiber. 1, 2, 3, and 4 represent the positions shown by magnification.

In response to the shortcomings in reference [23] (the parameters of MS-SMF are not suitable and the preparation method of ZIF-8/PDMS-sensitive film is not perfect), this paper has made improvements in the preparation of the MMI structure fiber and sensitive film. When reducing the cladding diameter of the MS-SMF, the core diameter remains invariable. The MMI structure fiber is fabricated by fusion and splicing technology, as shown in Figure 6. Firstly, a portion of MMF1 was spliced with the SMF1 by the fusion splicer and cleaved about 1 mm long with a small margin of error by using a high-precision cleaver, as shown in Figure 6a. The diameters of the MMF1 cladding and core are 125 µm and 62.5 µm, respectively. Secondly, one end of the MS-SMF is fused and spliced with the other end of the MMF1, and the MS-SMF is cut to the designed length using a microscope-assisted cleaver, as shown in Figure 6b. Thirdly, the end of the MS-SMF is spliced to the MMF2 with the fiber fusion splicer, as shown in Figure 6c. In order to guarantee that the fabricated multimode interference structure has good interference fringes, we used an amplified spontaneous emission light source (ASE) and an Optical Spectrum Analyzer (OSA) to monitor the transmission spectrum of the interferometer before the third splicing. Finally, we cascaded the SMF2, and the whole sensor is fabricated successfully, as illustrated in Figure 6d. A low-loss and high-strength intermodal interference structure fiber can be obtained by adjusting fiber fusion parameters, as shown in Figure 7a. A sensitive film solution is prepared by mixing ZIF-8 (purchased from Shanghai Yien Chemical Technology Co., Ltd., Shanghai, China), PDMS, n-hexane, vinyltriethoxysilane (VTES), and dibutyltin dilaurate (DBTDL). N-hexane helps to evenly mix various raw materials and reduce the viscosity of the mixed solution, making it easier for subsequent film coating. VTES is a silane coupling agent that can introduce silicon oxygen bonds during the preparation of PDMS, enhancing the cross-linking structure and heat resistance of PDMS. VTES can also improve the surface properties of PDMS and enhance its wettability and adhesion. DBTDL is an organotin compound that can be used as a catalyst for PDMS. During the preparation process of PDMS, DBTDL can promote the formation of silicon oxygen bonds and cross-linking reactions, accelerate the curing speed of PDMS, and improve its heat resistance. The ZIF-8/PDMS-sensitive film is coated on the surface of the MS-SMF cladding by the dip coating method. Figure 7b is the scanning electron microscopic (SEM) view of the cross-section of fiber coated with ZIF-8/PDMS-sensitive film. It can be seen that the thickness of the ZIF-8/PDMS-sensitive film is relatively uniform, with a thickness of about 1.8 μm.

**Figure 8:**
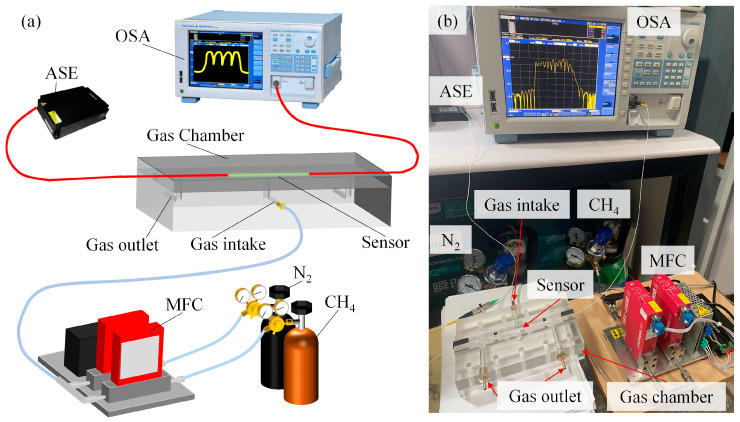
(**a**) Schematic diagram of the methane-sensing experiment test platform. (**b**) Physical diagram of the methane-sensing experiment test platform.

The performance of the sensor is tested by the methane-sensing test system. The methane-sensing test system is composed of a methane-mixing intake system and a sensing optical path, as shown in Figure 8. In the experiment, firstly, the methane sensor is fixed in the center of the gas chamber to ensure that the sensor is in full contact with methane gas. Then, the mass flow controller (MFC) is used to connect the methane cylinder and nitrogen cylinder, respectively. By controlling the output flow of methane and nitrogen, the mixing ratio of methane and nitrogen can be accurately controlled, so as to generate methane mixtures with different concentrations. Finally, the configured methane mixture is introduced into the gas chamber and the interference spectra at different methane concentrations are recorded by the OSA. For safety reasons, the concentration ranges of methane are set as 0–4%.

### 3.2. Methane Response Characteristics of the Sensor

**Figure 9:**
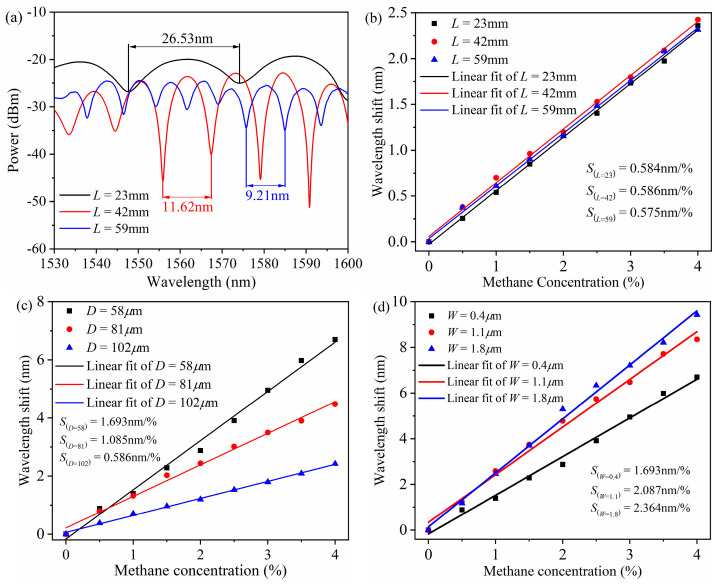
(**a**) Interference spectrum of methane sensor with MS-SMF length of 23 mm, 42 mm, and 59 mm. (**b**) Linear fitting diagram of the interference valley shits as methane concentration changes when MS-SMF length of 23 mm, 42 mm, and 59 mm. (**c**) Linear fitting diagram of the interference valley shifts as methane concentration changes for MS-SMF diameters of 58 µm, 81 µm, and 102 µm. (**d**) Linear fitting diagram of the interference valley shifts as methane concentration changes for sensitive film thicknesses of 0.4 µm, 1.1 µm, and 1.8 µm.

In the experiment of the effect of the MS-SMF length on the sensitivity, the sensor samples with an MS-SMF length of 23 mm, 42 mm, and 59 mm are prepared. As shown in Figure 9a, the FSR decreases with the increase in the MS-SMF length, but there is no significant difference in sensor sensitivity at different MS-SMF lengths, as shown in Figure 9b. In the experiment of the influence of the MS-SMF diameter on sensitivity, sensor samples with an MS-SMF diameter of 58 µm, 81 µm, and 102 µm are prepared. As shown in Figure 9c, the sensor sensitivity increases with the decrease in the MS-SMF diameter. When the diameter is 58 µm, the sensitivity reaches the maximum value, which is 1.693 nm/%. The sensor samples with a sensitive film thickness of 0.4 µm, 1.1 µm, and 1.8 µm are prepared in the experiment of the influence of sensitive film thickness on sensitivity. As shown in Figure 9d, the sensor sensitivity increases with the increase in the sensitive film thickness. When the thickness is 1.8 µm, the sensitivity reaches the maximum value, which is 2.364 nm/%, with a detection limit of 338 ppm. If the thickness of the sensitive film is too large, the increase in sensor sensitivity will tend to be stable, because the thicker sensitive film will not adsorb more methane molecules. On the contrary, it will affect the overall refractive index of the MS-SMF cladding, so it cannot continue to improve the sensitivity. When the thickness of the sensitive film is in the range of 1.5–2.0 µm, the sensitivity reaches the maximum value. The experimental results verify the correctness of the theoretical modeling and lay the foundation for the optimal design of the sensor.

**Figure 10:**
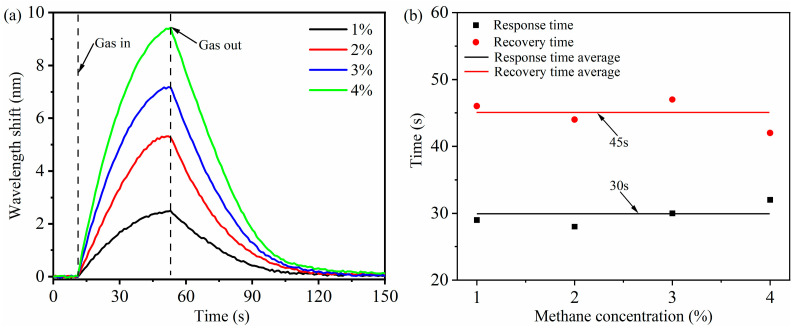
(**a**) Dynamic response of the methane sensor to 1–4% concentration. (**b**) Response and recovery time of the methane sensor at 1–4% concentration.

By controlling the concentration of methane entering the gas chamber, the response recovery curve of methane concentration in the range of 1–4% is measured, as shown in Figure 10. The results show that the sensor with the ZIF-8/PDMS-sensitive film has good response and recovery time. According to Figure 10a, a 90% fluctuation of response and recovery time can be obtained, as shown in Figure 10b, with an average response time of 30 s and recovery time of 45 s. The response speed is faster than the recovery speed, indicating that the adsorption performance of the ZIF-8/PDMS-sensitive film is better than the desorption performance.

### 3.3. Temperature Characteristics of Methane Sensor

**Figure 11:**
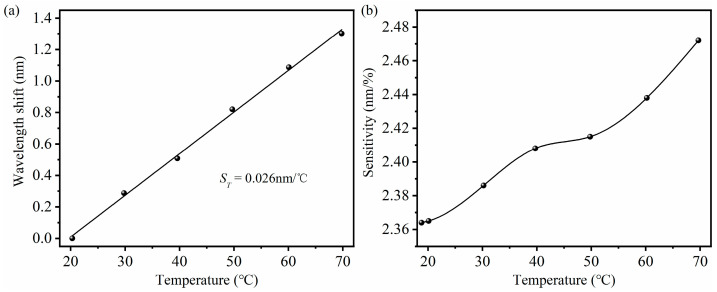
(**a**) Temperature sensitivity of methane sensor. (**b**) Sensitivity of methane sensors at different temperatures.

The room temperature is about 18.8 °C. In order to study the temperature response of methane sensors, the sensors are placed in a heating furnace and keep stationary during the experiment, heating the temperature from 20 °C to 70 °C in steps of about 10 °C. The temperature is changed by the heating furnace and monitored in real-time using a platinum resistance temperature sensor. Based on the thermal optical effect and thermal expansion effect of the material, the methane sensor responds to temperature changes. Figure 11a shows that as the temperature increases, the wavelength shift of the interference spectrum increases. The temperature sensitivity of the methane sensor is approximately 0.026 nm/°C. The sensitivity of methane sensors at different temperatures is shown in Figure 11b. As the temperature increases, the adsorption of methane by the sensitive membrane enhances, resulting in an increase in the sensitivity of the sensor. The sensitivity of the methane sensor is about 2.472 nm/% at a temperature of 70 °C, which is 4.57% higher than the sensitivity at room temperature. According to the temperature characteristics of methane sensors, it can be concluded that the impact of temperature changes on methane sensing can be ignored under usage conditions with a relatively small temperature variation range. When methane sensors are used in situations with a large range of temperature changes, the temperature characteristic curve of the methane sensor can be used to perform appropriate temperature compensations on the methane sensor.

Table 2 shows the experimental performance comparison between the sensor in this paper and other optical fiber methane sensors. Compared with other optical fiber methane sensors, the sensor proposed in this paper has advantages in terms of LOD, sensitivity, response, and recovery time. The proposed methane sensor may exhibit a great potential in fields requiring methane detection due to its simple structure, cost-effectiveness, and compact size.

## 4. Conclusions

In this paper, a multimode interference methane sensor based on a ZIF-8/PDMS composite membrane is studied, and its great potential in the field of methane detection is demonstrated. The sensitive mechanism, structure design, and sample test of the methane sensor are discussed systematically in this paper. The sensor realizes the detection of methane concentration by using the sensitive characteristics of multimode interference and the ZIF-8/PDMS composite membrane to methane. By optimizing the parameters of the MS-SMF length, diameter, and sensitive film thickness, the optimized multimode interference methane sensor using the ZIF-8/PDMS-sensitive film achieves a sensitivity of 2.364 nm/% at methane concentrations of 0–4%. The LOD is about 338 ppm. The average response time is 30 s and recovery time is 45 s. The temperature sensitivity of the methane sensor is approximately 0.026 nm/°C. The above performance effectively verifies that the proposed sensor has the characteristics of high sensitivity, simple manufacturing, and compact structure. The sensor has wide application potential in coal mine and other gas detection scenarios.

## Figures and Tables

**Table 1:** MMI fiber structure simulation model parameters.

Parameter	Value	Unit
MS-SMF core diameter	9	µm
MS-SMF cladding diameter	80	µm
MS-SMF length	50	mm
MMF1 and MMF2 core diameter	62.5	µm
MMF1 and MMF2 cladding diameter	125	µm
MMF1 and MMF2 length	1	mm

**Table 2:** Experimental performance comparison between the sensor in this paper and other optical fiber methane sensors.

Configuration	Sensitivity (nm/%)	LOD (ppm)	Measuring Range (%)	Response/Recovery Time(s)	Reference
LPG-SPR	0.344	-	0–3.5	50/65	[19]
PCF-SPR	1.99	-	0–3.5	-	[20]
PCF-MC	1.673	697.35	0–5	-	[21]
PCF-LPG	0.85	-	0–3.5	-	[22]
PCF-NCF	0.514	1600	0–3.5	60/180	[23]
PCF-MI	0.231	-	0–4	40/180	[24]
MMI	1.078	1800	0–3.5	60/180	[25]
MZI-MMI	2.364	338	0–4	30/45	This work

## Data Availability

The data that support the findings of this study are available from the corresponding authors upon reasonable request.

## Abbreviations

The following abbreviations are used in this manuscript:

SPR	Surface plasmon resonance
LPG	Long-period grating
MMI	Multimode interference
LOD	Limit of detection LOD
MOF	Metal-organic frameworks
ZIF-8	Zeolitic imidazolate framework-8
PDMS	Polydimethylsiloxane
MS-SMF	Methane-sensitive single mode fiber
MZI	Mach–Zehnder interferometer
MMF	Multimode fiber
SMF	Single mode fiber
FSR	Free spectral range
VTES	Vinyltriethoxysilane
DBTDL	Dibutyltin dilaurate
SEM	Scanning electron microscopic
ASE	Amplified spontaneous emission
OSA	Optical spectral analyzer
MFC	Mass flow controller
NCF	No-core Fiber
MI	Modal interference
